# MiR-126 and miR-126* regulate shear-resistant firm leukocyte adhesion to human brain endothelium

**DOI:** 10.1038/srep45284

**Published:** 2017-03-30

**Authors:** Camilla Cerutti, Laura J. Edwards, Helga E. de Vries, Basil Sharrack, David K. Male, Ignacio A. Romero

**Affiliations:** 1 Department of Life, Health and Chemical Sciences, Biomedical Research Network, Open University, Walton Hall, Milton Keynes, MK7 6AA, UK; 2 Randall Division of Cell and Molecular Biophysics, King’s College London, New Hunt’s House, Guy’s Campus, London SE1 1UL, UK; 3 Nottingham University Hospitals, QMC, Queen’s Medical Centre, Nottingham, NG7 2UH, UK; 4 Division of Rehabilitation Medicine, Royal Derby Hospital, Uttoxeter Road, Derby DE22 3NE, UK; 5 Department of Molecular Cell Biology and Immunology, Neuroscience Campus Amsterdam, VU University Medical Center, 1007 MB Amsterdam, the Netherlands; 6 Department of Neuroscience, Sheffield University, 385a Glossop Road, Sheffield, S10 2HQ, UK

## Abstract

Leukocyte adhesion to brain endothelial cells, the blood-brain barrier main component, is a critical step in the pathogenesis of neuroinflammatory diseases such as multiple sclerosis (MS). Leukocyte adhesion is mediated mainly by selectins, cell adhesion molecules and chemokines induced by pro-inflammatory cytokines such as TNFα and IFNγ, but the regulation of this process is not fully clear. This study investigated the regulation of firm leukocyte adhesion to human brain endothelium by two different brain endothelial microRNAs (miRs), miR-126 and miR-126*, that are downregulated by TNFα and IFNγ in a human brain endothelial cell line, hCMEC/D3. Using a leukocyte adhesion *in vitro* assay under shear forces mimicking blood flow, we observed that reduction of endothelial miR-126 and miR-126* enhanced firm monocyte and T cell adhesion to hCMEC/D3 cells, whereas their increased expression partially prevented THP1, Jurkat and primary MS patient-derived PBMC firm adhesion. Furthermore, we observed that miR-126* and miR-126 downregulation increased E-selectin and VCAM1, respectively, while miR-126 overexpression reduced VCAM1 and CCL2 expression by hCMEC/D3 cells, suggesting that these miRs regulate leukocyte adhesion by modulating the expression of adhesion-associated endothelial mRNA targets. Hence, human brain endothelial miR-126 and miR-126* could be used as a therapeutic tool to reduce leukocyte adhesion and thus reduce neuroinflammation.

Leukocyte trafficking from the blood into the central nervous system (CNS) is a multistep process^1^, where firm adhesion between leukocytes and brain endothelial cells forming the blood-brain barrier is a critical step both in immunosurveillance^2^ and in neuroinflammatory diseases such as multiple sclerosis (MS)^3^. In the CNS, leukocyte adhesion occurs in postcapillary venules^4^ and is orchestrated by locally secreted pro-inflammatory cytokines^5^,^6^,^7^ such as TNFα and IFNγ, which induce expression of selectins, cell adhesion molecules and chemokines as E-selectin, vascular adhesion molecule 1 (VCAM1), chemokine (C-C motif) ligand 2 and 7 (CCL2 or MCP1 and CCL7 or MCP3)^8^. These key molecules are expressed in MS lesions^7^,^9^,^10^ and have been shown to mediate firm leukocyte adhesion^4^,^11^,^12^. However, the exact molecular control by human brain endothelial cells in the regulation of leukocyte adhesion remains to be fully understood.

MicroRNAs (miRs) are a class of highly conserved, non-coding RNA molecules (20–25 nucleotides), that modulate gene expression by repression of their target genes at the post-transcriptional level^13^. MiRs are key regulators of a vast number of biological processes and disorders, including MS^14^ and those regulating neurovascular function in inflammation^15^, such as regulation of cell adhesion molecules and leukocyte adhesion to human brain endothelium^12^,^16^.

Human miR-126 (also known as miR-126-3p) and its complement, miR-126* (also known as miR-126-5p and originally named miR-123) originate from the same precursor, and their locus is hosted by intron-7 of the *Egfl7* (epidermal grow factor-like domain 7) gene on chromosome 9. MiR-126 and miR-126* are amongst the most abundant miRs expressed in resting endothelium from different vascular beds^17^,^18^, including CNS endothelium^19^.

MiR-126 is a well-studied miR in vascular biology with a critical role in angiogenesis and vascular integrity^17^,^20^ and it was the first miR studied in the context of endothelial adhesion molecule regulation in inflammation^18^. In addition, miR-126 regulates adhesion of human promyelocytic cell (HL-60) and chronic myelogenous leukemia (LAMA84) cells to human umbilical vein endothelial cells (HUVEC) by targeting VCAM1^18^,^21^. MiR-126* appears less abundant than miR-126 in endothelium^17^,^22^. It has been shown to be implicated in erythropoiesis^23^, endothelial cell turnover^24^, cancer cell motility^25^,^26^,^27^, monocyte recruitment by breast cancer epithelial cells through increased production of miR-126* targets CXCL12 (stromal cell-derived factor 1 Sdf-1a), CCL2^28^ and it regulates leucocyte trafficking in lung by controlling ALCAM expression^29^.

In this study, we investigated the roles of miR-126 and miR-126* in the control of leukocyte adhesion to human brain endothelium. Because leukocyte recruitment and adhesion *in vivo* occur in a dynamic system dominated by the shear flow of the circulating blood on the endothelium, we used a flow based adhesion assay. We report that human brain endothelial miR-126 and miR-126* regulate shear-resistant firm monocyte, T cell, healthy- and multiple sclerosis-derived PBMC adhesion to a human brain endothelial cell line, hCMEC/D3. Furthermore, we observed that human brain endothelial miR-126 and miR-126* effects on leukocyte adhesion to hCMEC/D3 can be partially accounted for by its modulation of expression of adhesion-related targets, VCAM1, CCL2 and E-selectin.

## Results

### TNFα + IFNγ increase E-selectin ICAM1 and VCAM1 expression, enhance firm leukocyte adhesion and downregulate miR-126 and miR-126* expression in hCMEC/D3 cells

**Figure 1:**
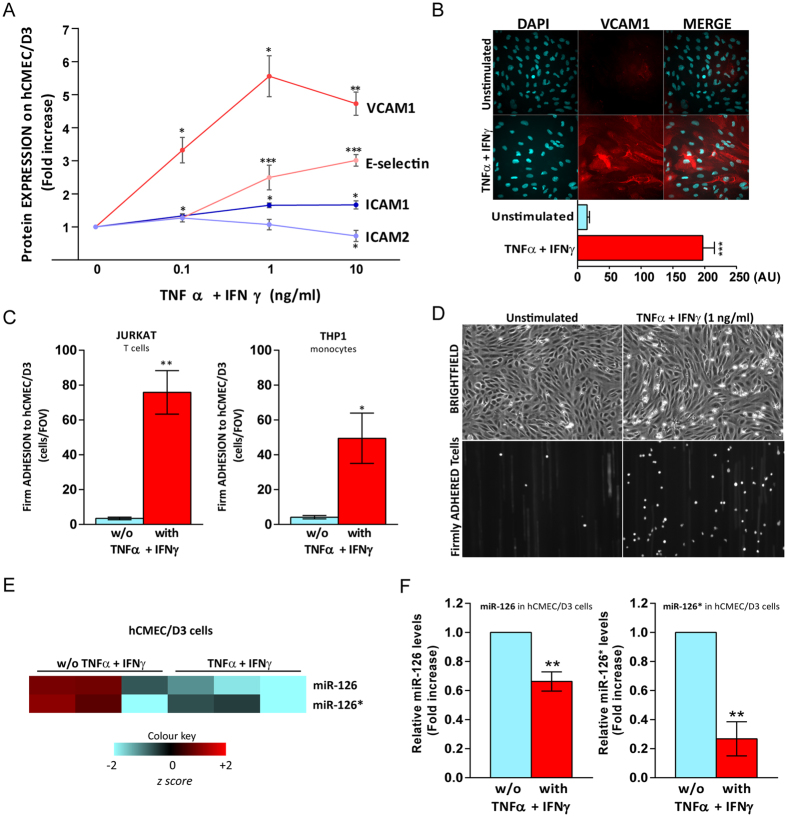
TNFα + IFNγ increase E-selectin, ICAM1 and VCAM1 expression, enhance firm leukocyte adhesion and downregulate miR-126 and miR-126* expression in hCMEC/D3 cells. hCMEC/D3 cell monolayers were treated with a combination of cytokines (TNFα + IFNγ) (**A**) at different concentrations (0, 0.1, 1, 10 ng/ml) or (**B**–**D**,**F**) at 1 ng/ml or left without (w/o) for 24 h. (**A**) VCAM1, ICAM1 and E-selectin expression levels were quantified by ELISA. (**B**) VCAM1 expression was quantified by immunofluorescence and expressed as Integrated Density in arbitrary units (A.U.). (**C**) Numbers of shear-resistant firmly adhered Jurkat and THP1 cells to hCMEC/D3 monolayer per field of view (FOV) (640 × 480 μm). (**D**) Representative images of shear-resistant firmly adhered fluorescently labelled Jurkat T cells (small white rounded cells) to unstimulated (left) or cytokine treated (right) hCMEC/D3 cells (monolayer of spindle shaped cells in bright field) per FOV (640 × 480 μm). (**E**) Heatmap representing miR-126 and miR-126* expression in unstimulated hCMEC/D3 cells and after stimulation (10 ng/ml of TNFα + IFNγ) for 24 h (n = 3). Individual repeats are shown in the heatmap. Blue indicates under expression, red overexpression, and intensity of color indicates relative change. Rows were colored using a z-score derived from a gene’s expression across all samples (row z-score). Data from Geo accession GSE21350 (Platform GPL14767) of miR-126 and miR-126* are represented. (**F**) Relative miR-126 and miR-126* level expression measured by qRT^2^-PCR from total RNA. The small nuclear RNA U6 was used as internal control. Experiments were carried out three times to four times with (**A**,**B**,**F**) three replicates (**C**) five FOV. Data are mean ± SEM. *P < 0.05, **P < 0.01, ***P < 0.001, *compared to untreated (w/o TNFα + IFNγ).

Leukocyte adhesion is mediated mainly by CAMs and selectins expressed by endothelium. Previous studies have shown that the expression of VCAM1 and ICAM1^30^ by hCMEC/D3 cells increased following stimulation with a combination of TNFα and IFNγ (100 U/ml + 100 ng/ml) for 24 h. In addition, it has been shown that TNFα alone increased E-selectin expression on primary human cerebral endothelium^31^. To assess the most suitable cytokine concentration to study leukocyte adhesion to human brain endothelial cells, a cytokine dose-response study on VCAM1, ICAM1 and E-selectin expression was performed by ELISA. An increase in VCAM1 (3-fold), ICAM1 and E-selectin (1.5-fold) expression by hCMEC/D3 cells was observed at the lowest concentration of cytokines used (0.1 ng/ml) (Fig. 1A). This effect was greater with 1 ng/ml (TNFα + IFNγ), but there was no further increase using higher concentration (10 ng/ml) of cytokines. ICAM2 is constitutively expressed on brain endothelium^32^ and does not increase in response to inflammatory stimuli^33^, thus ICAM2 was used as a control of a CAM whose levels are not increased by pro-inflammatory cytokines. Furthermore we observed a slight, but significant, decrease of ICAM2 expression levels by hCMEC/D3 cells when stimulated with cytokines at 1 ng/ml (Fig. 1A) as previously shown^34^. We confirm the effect of cytokines on VCAM1 expression on hCMEC/D3 monolayers cultured in the Ibidi^®^ μ-Slide VI^0.4^ by immunohistochemistry (Fig. 1B). Given the observed increases of CAM and selectin expression on cytokine-treated hCMEC/D3 cells at 1 ng/ml (TNFα + IFNγ), we next investigated leukocyte adhesion to hCMEC/D3 cells under shear stress using live cell imaging. We observed a striking increase of firmly adhered shear-resistant T cells (Jurkat) and monocytes (THP1) to cytokine-stimulated hCMEC/D3, when compared to unstimulated conditions (Fig. 1C,D). Previous results from our group indicate that TNFα and IFNγ in combination (10 ng/ml, 24 h) decreased miR-126 and miR-126* expression in hCMEC/D3 by microRNA array^35^, here we generate a heatmap for miR-126 and miR-126* expression using a z-score derived from gene’s expression across all samples (row z-score) from published data in the public database Gene Expression Omnibus (GEO)-Geo accession GSE21350) (Fig. 1E). To investigate whether lower concentration of cytokines still downregulate both miR-126 and miR-126* in hCMEC/D3 cells, and, to confirm the microRNA array data, we performed qRT^2^-PCR using U6 as housekeeping gene. We found that TNFα and IFNγ in combination (1ng/ml, 24 h) reduce both miR-126 and miR-126* relative levels in hCMEC/D3 cells when compared to their expression in unstimulated cells by 30 and 70%, respectively (Fig. 1F).

### Human brain endothelial miR-126 modulates THP1, Jurkat T cell, PBMC healthy donor and MS patient-derived firm adhesion to hCMEC/D3 cells

**Figure 2:**
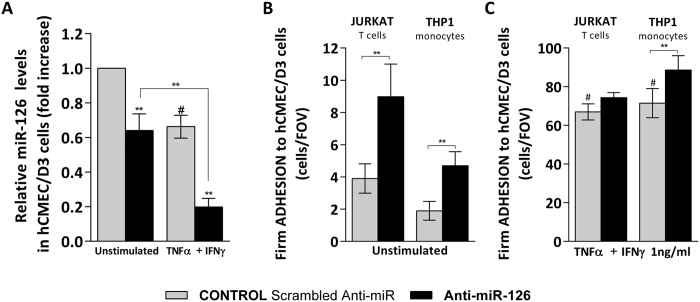
Human brain endothelial miR-126 downregulation enhances shear-resistant firmly adhered Jurkat and THP1 cells to hCMEC/D3 cells. hCMEC/D3 cell were transfected with control Scrambled Anti-miR (grey) or with Anti-miR-126 (black) followed by treatment with cytokines (TNFα + IFNγ, 1 ng/ml) for 24 h or left unstimulated. (**A**) Relative miR-126 level expression measured by qRT^2^-PCR from total RNA. The small nuclear RNA U6 was used as internal control. (**B**,**C**) Numbers of shear-resistant firmly adhered Jurkat T cells and THP1 monocytes to (**B**) unstimulated and (**C**) cytokine-stimulated hCMEC/D3 cells per field of view (FOV) (640 × 480 μm). Experiments were carried out three to five times (**A**) three replicates (**B**,**C**) five FOVs. Data are mean ± SEM. ^#^*P* < 0.05, **P < 0.01, ^#^compared to unstimulated.

To investigate whether human brain endothelial miR-126 regulates T cell and monocyte adhesion to hCMEC/D3 cells, we used a gain- and loss-of-function approach to modulate endogenous miR-126 expression in human brain endothelial cells. In order to mimic the effect of pro-inflammatory cytokines, we first downregulate the endogenous level of miR-126 in hCMEC/D3 cells using a miR-126 antagonist (anti-miR-126), a chemically modified antisense ssRNA that block the activity of endogenous miRs by complementarity. Brain endothelial cell transfection with anti-miR-126 significantly reduced miR-126 expression in unstimulated hCMEC/D3 cells when compared to control (scrambled Anti-miR), and it was further decreased in the presence of cytokines (Fig. 2A). Then, we investigated whether miR-126 downregulation mimics the inflammatory niche in the context of monocytic and T cell adhesion to hCMEC/D3 cells under shear stress. Transfection with anti-miR-126 increased firm T cell (Jurkat) and monocyte (THP1) adhesion to unstimulated hCMEC/D3 cells by 50% (Fig. 2B), this effect was maintained to a lesser extent in the presence of cytokine in firm monocyte adhesion, however no significant changes were observed in cytokine-induced T cell adhesion (Fig. 2C).

**Figure 3:**
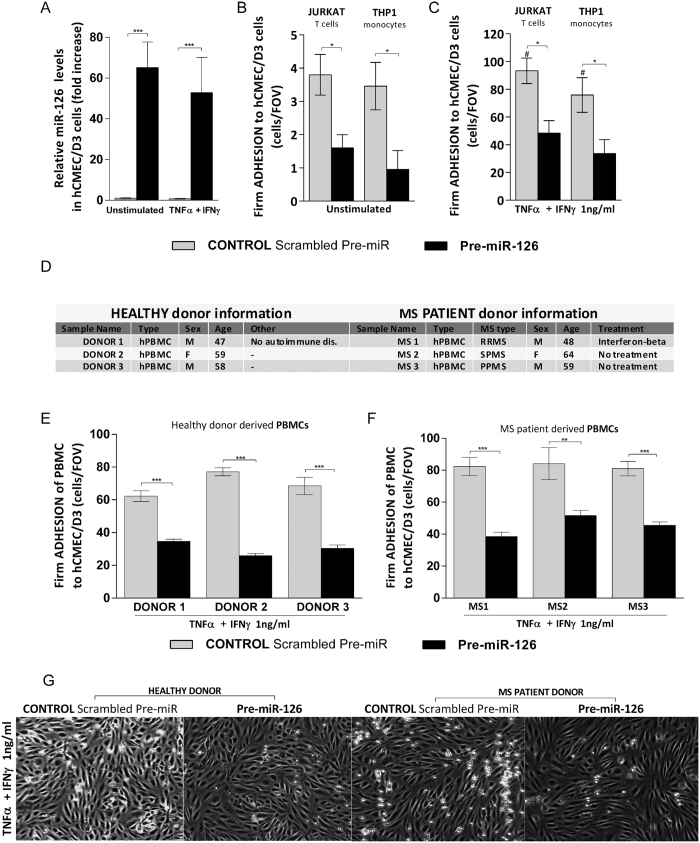
miR-126 upregulation decreases monocytic, T cell, PBMC and MS-derived PBMC firm adhesion to hCMEC/D3 cells. hCMEC/D3 cells were transfected with control Scrambled Pre-miR (grey) or with Pre-miR-126 (black) followed by treatment with cytokines (TNFα + IFNγ, 1 ng/ml) for 24 h or left unstimulated. (**A**) Relative miR-126 level expression measured by qRT^2^-PCR from total RNA. The small nuclear RNA U6 was used as internal control. (**B**,**C**) Numbers of shear-resistant firmly adhered Jurkat T cells and THP1 monocytes to (**B**) unstimulated and (**C**) cytokine-stimulated hCMEC/D3 monolayers per field of view (FOV) (640 × 480 μm). (**D**) Information of the three PBMC healthy donors and multiple sclerosis (MS) patient PBMC donors; pathological features, relapsing-remitting MS (RRMS), secondary progressive MS (SPMS), primary progressive (PPMS). (**E**,**F**) Numbers of shear-resistant firmly adhered (**E**) healthy donor PBMC and (**F**) MS patient derived PBMC to cytokine-stimulated hCMEC/D3 monolayers per field of view (FOV) (640 × 480 μm). (**G**) Representative images of shear-resistant firmly adhered healthy donor PBMC and MS patient derived PBMC MS to control scrambled Pre-miR or Pre-miR-126 transfected and cytokine-treated hCMEC/D3 cells (monolayer of spindle shaped cells in bright field) per FOV (640 × 480 μm). Experiments were carried out (**B**,**C**) three times to five times with five FOVs and (**E**,**F**) with ten FOVs. Data are mean ± SEM. *^,#^P < 0.05, **P < 0.01, ***P < 0.001, ^#^compared to unstimulated.

To counteract cytokine-induced miR-126 downregulation, which led to an increased firm leukocyte adhesion to the human brain endothelium, we increased endogenous miR-126 levels in hCMEC/D3 cells with miR-126 precursors (pre-miR-126), a synthetic dsRNA that simulates naturally occurring mature miR-126. Brain endothelial cell transfection with pre-miR-126 significantly increased miR-126 expression in hCMEC/D3 cells both in the presence and absence of cytokines when compared to control (scrambled Pre-miR) (Fig. 3A). Increased human brain endothelial miR-126 expression remarkably decreased shear resistant T cell and monocyte adhesion by almost 50% to both unstimulated (Fig. 3B) and cytokine-treated human brain endothelial cells (Fig. 3C). To further demonstrate miR-126 contribution to the considerable reduction of immune cell firm adhesion to human brain endothelial cells in the context of vascular neuroinflammation, we reassessed the effect of miR-126 upregulation in cytokine-stimulated hCMEC/D3 cells with healthy donor and multiple sclerosis (MS) patient-derived peripheral blood mononuclear cell (PBMC) in order to mimic more closely the *in vivo* inflammatory event. Three different healthy donors and MS patient-derived PBMC from with different types of MS were used (Fig. 3D), relapsing-remitting MS (RRMS), secondary progressive MS (SPMS) and primary progressive (PPMS). We tested each healthy and MS donor sample for CD4+, CD8+, monocyte CD14+ and NK cells subpopulation proportion, and, we found they were comparable between samples (Supplemental Fig. S1). Using live cell adhesion imaging under flow conditions, we found that brain endothelial miR-126 overexpression decreased both healthy donor and MS-derived PBMC firm adhesion to cytokine-treated hCMEC/D3 cells by 50–60% in all samples tested (Fig. 3E,F and supplemental Video S1) when compared to control (Fig. 3E,F and supplemental Video S2). This confirms that miR-126 plays a role in the regulation of immune cell adhesion to human brain endothelium. In addition, we observed that MS derived PBMC adhesion to hCMEC/D3 cells under shear stress was initially characterized by capture of individual single cells, followed by, in some areas, branched strings formation of adhered PBMC around the previously captured cells, as shown in Fig. 3G and most of them were CD8+ T cells (Supplemental Fig. S1) as previously reported^36^, and, recently confirmed^37^.

### Human brain endothelial miR-126* modulates leukocyte firm adhesion to hCMEC/D3 cells

**Figure 4:**
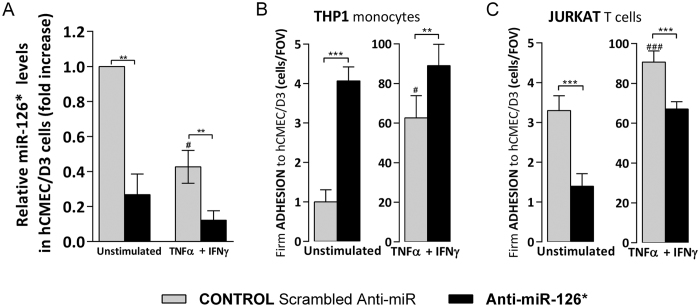
miR-126* downregulation regulates THP1 monocyte and Jurkat T cell firm adhesion to hCMEC/D3 cells. hCMEC/D3 cell were transfected with control Scrambled Anti-miR (grey) or with Anti-miR-126* (black) followed by treatment with cytokines (TNFα + IFNγ, 1 ng/ml) for 24 h or left unstimulated. (**A**) Relative miR-126* level expression measured by qRT^2^-PCR from total RNA. The small nuclear RNA U6 was used as internal control. (**B**,**C**) Numbers of shear-resistant firmly adhered (**B**) THP1 monocytes and (**C**) Jurkat T cells to unstimulated and cytokine-stimulated hCMEC/D3 cells per field of view (FOV) (640 × 480 μm). Experiments were carried out three times with (**A**) two replicates (**B**,**C**) five FOVs. Data are mean ± SEM. ^#^*P* < 0.05, **P < 0.01, ***^,###^*P* < 0.001, ^#^compared to unstimulated.

**Figure 5:**
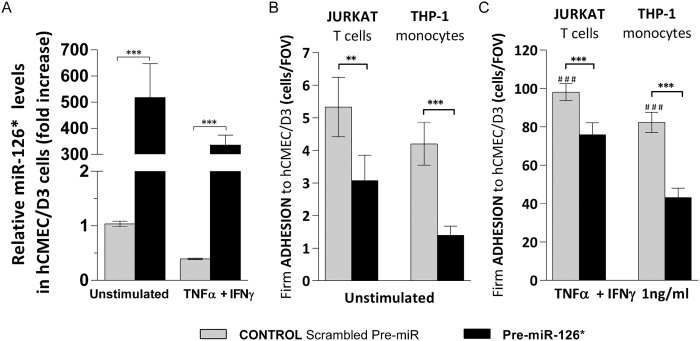
miR-126* upregulation decreases shear-resistant firmly adhered Jurkat and THP1 cells to hCMEC/D3 cells. hCMEC/D3 cells were transfected with control Scrambled Pre-miR (grey) or with Pre-miR-126 (black) (**A**) Relative miR-126* level expression measured by qRT^2^-PCR from total RNA. The small nuclear RNA U6 was used as internal control. (**B**,**C**) Numbers of shear-resistant firmly adhered THP1 monocytes and Jurkat T cells to (**B**) unstimulated and (**C**) cytokine-stimulated hCMEC/D3 cells per field of view (FOV) (640 × 480 μm). Experiments were carried out three times with five FOVs. Data are mean ± SEM. ^#^*P* < 0.05, **P < 0.01, ***^,###^*P* < 0.001, ^#^compared to unstimulated.

MiR-126 partial complement, miR-126*, is also downregulated by cytokine (Fig. 1F) and by transfection with anti-miR-126* in unstimulated and cytokines-treated hCMEC/D3 cells (Fig. 4A). MiR-126* downregulation increased monocyte firm adhesion to both unstimulated and cytokine-stimulated hCMEC/D3 cells (Fig. 4B), while Jurkat T cell adhesion was, unexpectedly, partially prevented (Fig. 4C). The difference in miR-126* effect on monocyte and T cell firm adhesion could be accounted by its effect on different adhesion and immune-specific target mRNA of proteins that mediate adhesion. Increased levels of endothelial miR-126* with pre-miR-126* (Fig. 5A) still decreased both Jurkat T (~20%), THP1 (~50%) firm adhesion either to unstimulated (Fig. 5B) and cytokine-stimulated (Fig. 5C) hCMEC/D3 cells under flow. Furthermore, pre-miR-126* decreased healthy donor derived PBMC adhesion to stimulated hCMEC/D3 cells under flow (Fig. S2). These findings suggest that monocyte and T cell adhesion to brain endothelium may be regulated via different intracellular mechanisms by miR-126*.

### Human brain endothelial miR-126 and miR-126* target different adhesion-related genes

**Figure 6:**
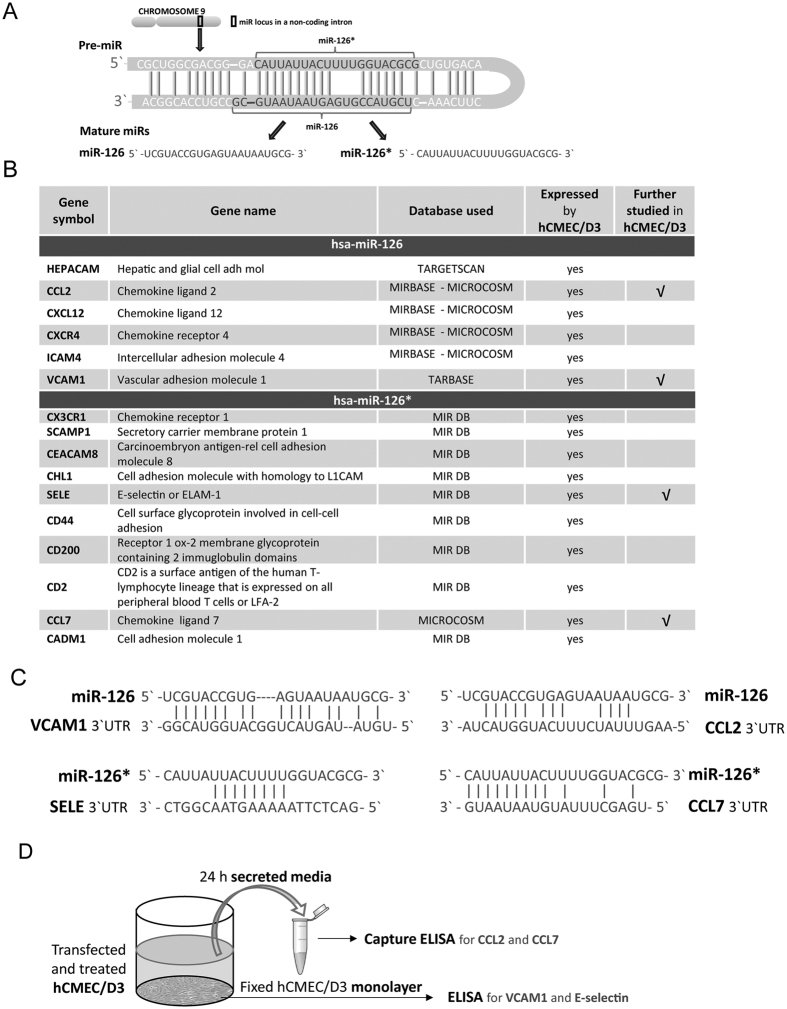
Identification of miR-126 and miR-126* adhesion-related putative gene targets *in silico*. (**A**) Stem loop of the primary precursor (Pri-miR) structure of human miR-126 and miR-126* and the mature hsa-miR-126 and has-miR-126* sequences. (**B**) List of adhesion-associated hsa-miR-126 and hsa-miR-126* predicted targets *in silico*. (**C**) Sequence alignment of the predicted duplex formation between mature miRs and their putative gene target mRNA selected for further study in hCMEC/D3 cells. (**D**) Graphic representation of the experimental design used to study the predicted target at protein level, the same experimental well was used to study membrane associated protein and secreted protein.

**Figure 7:**
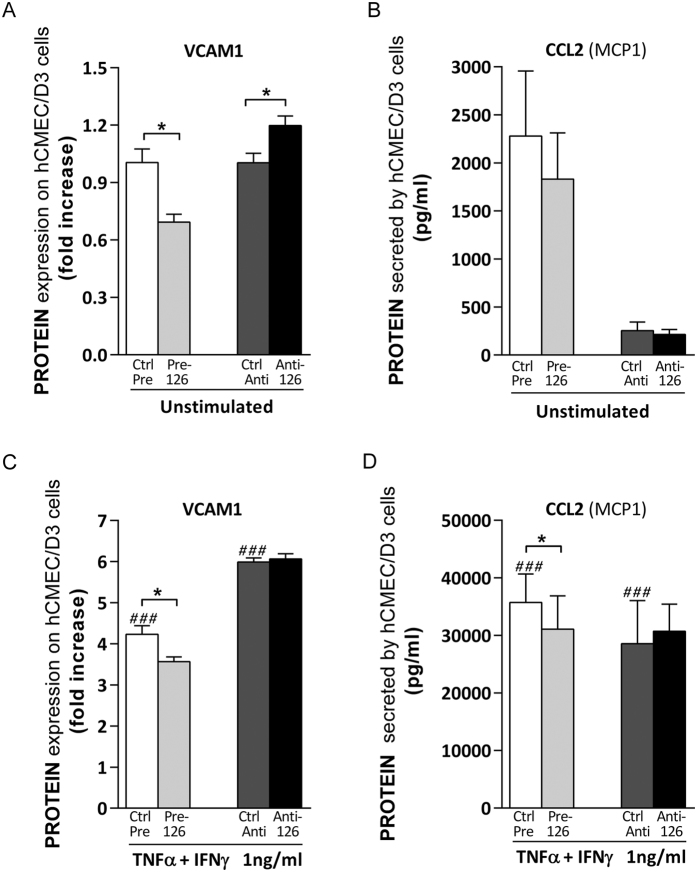
Brain endothelial miR-126 modulates VCAM1 and CCL2 in hCMEC/D3 cells. (**A**–**D**) hCMEC/D3 cells were transfected with control Scrambled Pre-miR or Pre-miR-126 or control Scrambled Anti-miR or Anti-miR-126 (**C,D**) followed by treatment with cytokines (TNFα + IFNγ, 1 ng/ml) for 24 h or (**A**,**B**) left unstimulated. (**A,C**) Membrane-associated VCAM1 expression was quantified by ELISA and (**B**,**D**) secreted CCL2 (MCP1) was measured by capture ELISA. Experiments were carried out (**A**,**C**) four or (**B**,**D**) three times with three replicates each. Data are mean ± SEM. *P < 0.05, ^###^*P* < 0.001, ^#^compared to unstimulated.

To further elucidate the role of miR-126 and miR-126*, in the regulation of shear resistant monocyte, T cell and PBMC adhesion to brain endothelial cells, we explored possible molecular mediators for this observed biological response among miR-126 and miR-126* targets. Human mature miR-126 and miR-126* originate from the same primary transcript (Pre-miR) and their sequences are different in base composition (Fig. 6A), miR-126* (hsa-miR-126-5p mature sequence CAUUAUUACUUUUGGUACGCG and miRbase accession MIMAT0000444) and miR-126* (hsa-miR-126-3p mature sequence UCGUACCGUGAGUAAUAAUGCG and miRbase accession MIMAT0000445), thereby targeting different genes. First, we systematically collated miR-126 and miR-126* predicted targets involved in leukocyte adhesion using databases available on-line (listed in methods) to predict target gene transcripts *in silico.* Then, we selected adhesion related-targets previously found to be involved in neuroinflammatory diseases and/or leukocyte trafficking, and, that are expressed by hCMEC/D3 (Geo: GSM1089040)^35^ (Fig. 6B). Finally, we selected for further studies, four of these proteins that are master mediators of leukocyte trafficking: miR-126 predicted targets VCAM1 and CCL2 (MCP1), and miR-126* predicted targets SELE (E-selectin) and CCL7 (MCP3) (Fig. 6C). Although VCAM1 and CCL2 have been previosly shown to be miR-126 gene targets in different endothelial and non-endothelial cell types^18^,^21^,^38^,^39^,^40^,^41^,^42^,^43^,^44^, it has not been reported in human brain endothelium. We tested the effects of miR-126 and miR-126*, using gain-and loss-of-function approach, on their mRNA expressed in hCMEC/D3 cells respective targets at the protein level following the experimental design depicted in Fig. 6D. Decreased or increased levels of miR-126 in unstimulated hCMEC/D3 cells significantly enhanced or reduced membrane-associated VCAM1 expression respectively (Fig. 7A), while no significant changes in secreted endothelial CCL2 were detected (Fig. 7B). Cytokines-treatment (TNFα and IFNγ) strongly increased membrane-associated VCAM1 level and CCL2 secretion by brain endothelial cells (Fig. 7C,D) compared to unstimulated (Fig. 7A,B), while miR-126 upregulation slightly decreases the cytokine effects on hCMEC/D3 cells with reduction in VCAM1 expression (Fig. 7C) and CCL2 secretion (Fig. 7D). This finding correlates with the effect of miR-126 on both monocytic and T cell adhesion to human brain endothelial cells shown in Fig. 3.

**Figure 8:**
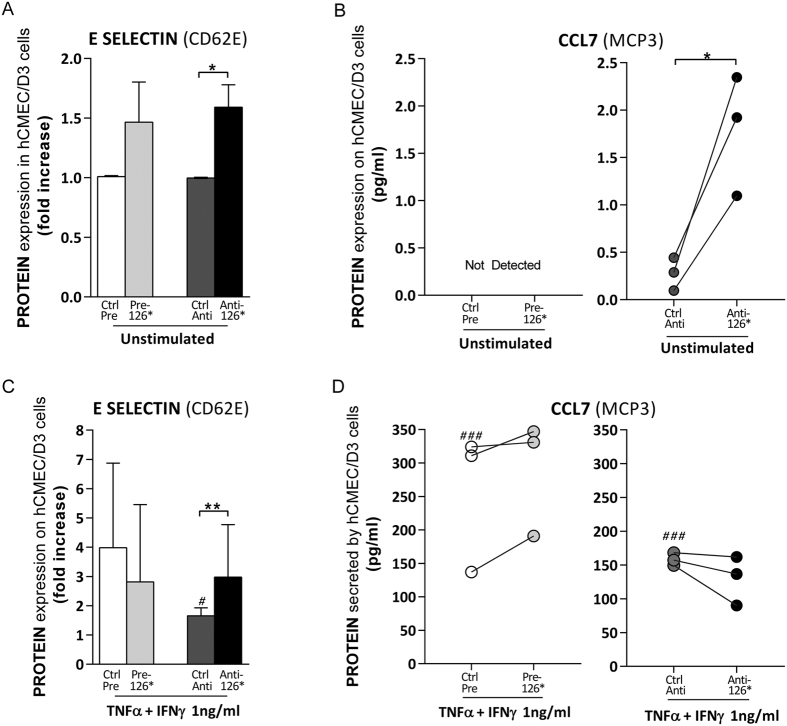
Brain endothelial miR-126* modulates E-selectin in hCMEC/D3 cells. (**A–D**) hCMEC/D3 cells were transfected with control Scrambled Pre-miR or Pre-miR-126 or control Scrambled Anti-miR or Anti-miR-126 (**C**,**D**) followed by treatment with cytokines (TNFα + IFNγ, 1 ng/ml) for 24 h or (**A**,**B**) left unstimulated. (**A**,**C**) Membrane-associated E-selectin (CD62E) expression was quantified by ELISA and (**B**,**D**) secreted CCL7 (MCP3) was measured by capture ELISA. Experiments were carried out three times with three replicates each. Data are mean ± SEM. *P < 0.05, **P < 0.01, ^###^*P* < 0.001, ^#^compared to unstimulated.

Brain endothelial miR-126* downregulation increases membrane-associated E-selectin (CD62E) expression (Fig. 8A) and CCL7 secretion (Fig. 8B) in unstimulated hCMEC/D3 cells. In cytokine (TNFα and IFNγ) treated brain endothelial cells, anti-miR-126* treatment induced a small increase of membrane-associated E-selectin expression (Fig. 8C), however no significant difference in CCL7 secretion was observed when compared to control (control-anti-miR) (Fig. 8D). No changes in E-selectin and CCL7 expression were detected in pre-miR-126*-transfected hCMEC/D3 cells (Fig. 8A–D). Interestingly, we found that unstimulated hCMEC/D3 cells secreted less than 15.6 pg/ml CCL7, the lowest detectable amount above the assay’s threshold (Fig. 8B). Furthermore, we observed that following modulation of miR-126* levels in cytokine-treated hCMEC/D3 cells, there is a tendency to modulate CCL7 secretion (Fig. 8D). It is possible that differences in modulation of E-selectin and CCL7 expression by miR-126* contribute to the different behaviour in T cell and monocyte adhesion to anti-miR-126*-transfected hCMEC/D3 cells (Fig. 4B,C).

Taken together, our results indicate that brain endothelial miR-126 and miR-126* regulate monocytic and T cell shear-resistant adhesion to human brain endothelial cells, partially, via modulation of VCAM1, CCL2, E selectin and CCL7 target genes.

## Discussion

Leukocyte adhesion at the blood-brain barrier is believed to be a critical step in leukocyte extravasation that triggers neuroinflammatory diseases such as MS, characterized by a dramatic increase of pro-inflammatory cytokines such as TNFα and IFNγ^45^, which induce endothelial E-selectin, ICAM1, VCAM1, CCL2 (MCP1) and CCL7 (MCP3) expression and modulate leukocyte adhesion to the endothelium^30^,^31^,^46^. Here, we show that TNFα and IFNγ in combination, significantly increased E-selectin, ICAM1, VCAM1, CCL2 and CCL7 expression on hCMEC/D3 cells, using a lower dose of cytokines (1ng/ml) than previously (10–100 ng/ml) in order to avoid apoptosis in brain endothelial cells^47^. Using live-cell imaging, we investigated shear resistant firm adhesion of leukocytes to brain endothelium under continuous flow, that permits to study only specific adhesion and avoids unspecific cell-cell binding and/or interaction, and mimics leukocyte adhesion *in vivo*. Firm adhesion to cytokine-stimulated hCMEC/D3 cells was increased, as expected, furthermore we observed that the number of firmly adhered Jurkat T cells was higher than THP1 monocytes, consistent with the ratio of T cells/monocytes found in the perivascular infiltrate in MS active lesions^9^ and dependent on Jurkat and THP1 chemokine receptors, integrin and surface glycation profile.

New therapies, such as Natalizumab, have been developed to physically prevent the interaction between VLA-4 (α4-integrin) expressed by T cells and VCAM1 expressed by brain endothelium. However, these therapies may lead to progressive multifocal leukoencephalopathy^48^. Recently, miRs have been shown to represent possible therapeutic targets that act endogenously at the post-transcriptional level regulating a large number of different mRNA targets (members of distinct signaling pathways) modulating their expression^49^,^50^, although quantitatively the changes are often individually small^51^, a single miR can significantly impact a number of target genes directly and/or indirectly, so it can have a profound impact on complex cellular processes, by modulating several proteins involved in one process^52^.

In this study we show that treatment with TNFα and IFNγ (1 ng/ml) for 24 hours downregulates endogenous levels of human brain endothelial miR-126 and miR-126*. MiR-126 and miR-126* were found down-regulated in different inflammatory-associate diseases such as cystic fibrosis^53^, ischaemia^54^, atherosclerosis^24^, stroke^43^, and, in melanoma, breast and lung cancer cells^27^,^42^,^55^. In this paper, we specifically addressed the regulation of shear resistant leukocyte adhesion to brain endothelium by brain endothelial miR-126 and miR-126*. Although, miR-126 has been shown to regulate HL-60 cell adhesion to HUVEC^18^, and, in the context of CNS, breast cancer miR-126 and miR-126* repress recruitment of mesenchymal stem cells and inflammatory monocytes to inhibit breast cancer metastasis in brain^42^, the role of brain endothelial miR-126 and miR-126* in inflammation at the human neurovascular unit and/or in multiple sclerosis has not been investigated. We established that human brain endothelial miR-126 and miR-126* regulate firm leukocyte adhesion to hCMEC/D3 cells, and in particular, that increased miR-126 and miR-126* levels in brain endothelial cells, significantly reduced leukocyte adhesion, not only in untreated cells, but also in the presence of proinflammatory cytokines.

There are two possible explanations for these findings: the first one is that the cytokines cause a direct reduction in the expression of miR-126 and miR-126* which allows a consequent increase in the half-life of mRNA for selectins, adhesion molecules and chemokines, in turn leading to increased protein expression. Alternatively, the second is that cytokine-induced mRNA for the selectins, adhesion molecules and chemokines may lead to a consumption of miRs that target those mRNAs.

The first mechanism is a primary effect caused directly by cytokine activation of the cells, and the second mechanism is a consequence of the induction of other genes. Control of the *Egfl7* gene which includes the gene segments encoding for miR-126 and miR-126* is reported to be under the control of transcription factors Erg, GATA-2 and Ets-1/2^56^. Ets-1 is induced by TNFα^57^, so modulation of *Egfl7* and the miRs could follow TNFα stimulation. In turn, Ets-1 controls a wide range of cytokines and chemokines involved in neuroinflammation^58^. Meanwhile, it has been shown that the early (6 h) cytokine-induced miR-155, that contributes to regulate firm leukocyte adhesion^12^ to human brain endothelial cells and modulates VCAM1, ICAM1 expression^12^, targets the transcription factor Ets-1^59^. Indeed, endothelial Ets-1 down-regulation decreases transactivation of *Egfl7* and leads to miR-126/-126* down-regulation^56^, which may explain the decrease of endothelial miR-126 and miR-126* levels at 24 hours after cytokine stimulation (no significant changes were observed at 6 h, Geo accession GSE21350), thus after miR-155 upregulation by cytokines. Though, we think that the second explanation is more likely, because the decrease in miR-126 and miR-126* was seen 24 hours after cytokine stimulation, during which time mRNA for VCAM1, E selectin, CCL2 and CCL7 are induced by TNFα and IFNγ via either AP1 or NF-κB, as we observed increases in protein expression at 24 h and early time points (Figs 1,7,8 and S3) with relative increases in monocyte and T cell adhesion (Fig. S4). Furthermore, NF-κB controls gene transcription of miR-146a, another brain endothelial cytokine-induced miR that contributes to leukocyte adhesion regulation via repression of RhoA and NFAT5 leading to CCL2 and VCAM1 downregulation in hCMEC/D3 cells^16^. NF-κB cooperates with miR-155 targets and miR-126/-126* transcription factors Ets-1 to control miR-146a expression in T cell, then we can speculate that miR-126 and miR126* expression may also depend on miR-146a and miR-155 cytoplasmic levels. This may result from the fine tuning properties of miRs and the kinetics of molecules involved directly and indirectly in miR-126 and miR-126* regulation. Thus, the two mechanisms are not mutually exclusive, and both imply that miR-126 and miR-126* are involved in controlling the levels of some of the key molecules required for leukocyte migration into the CNS.

MiR-126 and miR-126* have different mature sequences, thus different gene targets motifs. As a result, we showed some differences in their actions on leukocyte adhesion to human brain endothelium. In particular, the firm adhesion of Jurkat cells was slightly decreased by treatment with anti-miR-126*, whereas THP1 responded by increased adhesion as anticipated. Here, we observed that miR-126* regulates E-selectin, for the first time. Endothelial E-selectin mediates T cell rolling and adhesion through PSGL-1 (P-selectin glycoprotein ligand-1)^60^ which is expressed by Jurkat T cells^61^, however while E-selectin expression increases on hCMEC/D3, Jurkat adhesion is reduced by anti-miR-126*. The best explanation for the difference may be that miR-126* targets other endothelial mRNA targets -not investigated here- that are modulating Jurkat T cell, but not THP1 monocytic adhesion. Indeed, although some miR-126* target genes have been investigated here, it is likely that the large effect observed on leukocyte adhesion by miR-126* modulation is the result of small effects on expression of many target genes (some listed in Fig. 6B), taking also in consideration that the miRs star form is less abundant^22^, thus the effect on a single target gene is smaller. A possible candidate is CD200, a predicted target of miR-126*, which is highly expressed in the CNS and involved in the pathology of MS, it has been shown to be involved in T cell-endothelium, but not in monocyte-endothelium interactions^62^. Furthermore, activated leukocyte cell adhesion molecule (ALCAM), a biologically validated target of miR-126* in HUVEC^29^, has been shown to be upregulated in neuroinflammation (multiple sclerosis lesions) and mediates leukocyte migration into the CNS, in particular CD14+ monocytes^63^.

We show that miR-126 modulates CCL2 and VCAM1 expression in hCMEC/D3 cells, in particular an increase of miR-126 decreases both VCAM1 and CCL2 in the presence of cytokines, which correlates with a decrease of THP1, Jurkat or PBMC (healthy or MS donor) firm adhesion to human brain endothelial cells. In particular, the results with MS patient-derived PBMC confirmed the possible therapeutic effect of pre-miR-126, in the context of human brain endothelial inflammation. Furthermore, the findings with VCAM1 are particularly relevant to CNS disease, since this adhesion molecule is highly regulated on human brain endothelium, by comparison with non-brain endothelium and the VLA-4/VCAM1 adhesion system is a proven target for clinical treatment of neuroinflammation.

The observation that individual miRs can affect several molecules involved in a single cellular process, and that miRs that originate from the same precursor modulate different gene targets, which act simultaneously on the same molecular event such as leukocyte adhesion, reinforces the concept that miRs may be suitable targets for anti-inflammatory therapy. MiR-based gene therapy for cancer and other non-brain diseases has been already approved in clinical trials^64^, although only for diseases that can be easily treated locally such as chronic asthma with miR-126 and liver cancer with miR-26a and miR-34^65^,^66^. *In vivo*, miR-126 was successfully delivered for therapeutic intervention in a mouse hind-limb ischemia model^67^, and miR-146a, a known miR that modulates T cell adhesion to human brain endothelium^16^, in aorta endothelium by micro particles via E-selectin^68^. However, there is need for optimization of miR delivery to brain endothelial cells *in vivo*.

In conclusion, our study demonstrates the roles of miR126 and miR126* in controlling leukocyte interaction with human brain endothelium under shear stress conditions. It also shows that exogenous modulation of these two different miRs in human brain endothelium can reduce the levels of monocyte and T cell firm-adhesion, which is the critical step determining leukocyte migration into the CNS. Our results imply that miR-126 and miR-126* are potential targets for anti-inflammatory therapies since they simultaneously modulate multiple proteins required for leukocyte adhesion.

## Methods

### Cell culture

The hCMEC/D3 cell line^30^ was used at passages 26–34 and cultured in endothelial cell basal medium-2 (EGM-2) medium (Lonza, Walkersville, USA) and supplemented with the following components obtained from the manufacturer: 0.025% (v/v) rhEGF, 0.025% (v/v) VEGF, 0.025% (v/v) IGF, 0.1% (v/v) rhFGF, 0.1% (v/v) gentamycin, 0.1% (v/v) ascorbic acid, 0.04% (v/v) hydrocortisone and 2.5% (v/v) foetal bovine serum (FBS), hereafter referred to as endothelial complete medium. hCMEC/D3 cells were grown to confluence (~1 × 10^5^ cells/cm^2^) on tissue culture flasks coated with collagen from calf skin (Sigma, St. Louis, USA). The T cell line Jurkat from acute T cell leukaemia and the monocytic line THP1 from acute monocytic leukaemia were a kind gift from Dr V Male (Cambridge University). Jurkat and THP1 cells were grown in suspension in RPMI 1640 W/GLUTAMAX I (Gibco^®^ Invitrogen, Paisley, UK) culture medium (with 10% FBS and 100 μg/ml streptomycin + 100 units/ml penicillin) and used in exponential growth for all experiments with no further treatment. All cell lines were maintained in a 95% humidified air and 5% CO_2_ incubator at 37 °C. PBMC from healthy donors (Stemcell Technologies, Cambridge, UK) were thawed, washed, resuspended in complete media, recovered for 4 hours and used with no further treatments. MS patients recruited by Dr. Giulio Podda and Dr. Bruno Gran during their routine consultations in the Neurology department at Nottingham University Hospitals NHS Trust. Blood samples were collected, transported, handled and used for the experiments following the protocols approved by the local research ethical committee at both Nottingham University Hospitals NHS Trust and The Open University (HREC/2011/#913, NRES 08/H0408/167), the approved human tissue transfer agreement and the signed informed consents were obtained from all blood donors. PBMC were isolated from fresh heparinised blood of three MS patients by density dependent centrifugation using Ficoll-Paque PLUS and frozen in 10% DMSO in liquid nitrogen until used for flow-based leukocyte adhesion assay, where PBMC were thawed, washed, resuspended, recovered for 4 hours in complete media and used with no further treatments. methods were carried out in accordance with the relevant guidelines and regulations.

### MicroRNA transfection

hCMEC/D3 cells were grown to ~70% confluence and transfected in antibiotic-free endothelial media. To introduce miR-126 or miR-126* precursor, hCMEC/D3 cells were transfected with 30 nM of pre-miR-126 or pre-miR-126* or its control, scrambled-pre-miR (Ambion, Fischer Scientific UK), using Siport™ Polyamine Transfection Agent (Ambion) in Opti-mem^®^ I (Gibco^®^) media for 24 h. For inhibition studies, 60 nM of anti-miR-126 or anti-miR-126* and its control, scrambled-anti-miR (Ambion, Fischer Scientific UK) were transfected using Lipofectamine^®^ 2000 (Thermo Fisher Scientific, Carlsbad, USA) for 6 h, media was then changed with endothelial complete medium for 18 h.

### Flow-based leukocyte adhesion assay: live cell adhesion imaging under flow conditions

A flow-based adhesion assay described previously in Cerutti *et al*.^12^ was used. hCMEC/D3 cells were grown in Ibidi^®^ μ-Slide VI^0.4^ (Ibidi^®^ GmbH, Martinstreid, Germany), transfected, treated with 1 ng/ml TNFα and IFNγ or left untreated for 24 h in static conditions and washed before flow adhesion assay. THP1 and Jurkat cells (2 × 10^6^cells/ml) were labelled with CMFDA (5-chloromethylfluoresceindiacetate, Life Technologies, Eugene, USA), while MS patient-derived PBMC were left unlabelled and were allowed to flow through the channel with endothelial monolayers and accumulate at 0.5 dyn/cm^2^ for 5 min. Then, the flow was increased to 1.5 dyn/cm^2^ (venular vessel wall shear stress) for 30 seconds to remove non-adhered leukocytes with endothelial complete media. The flow rate (θ) applied to produce the required shear stress τ (dyn/cm^2^) was calculated by Ibidi^®^ for the μ-slideVI 0.4 according to the equation τ [dyn/cm^2^] = η [(dyn * s)/cm^2^]∙176.1 Φ ml/min], where the relationship between shear stress (τ) and flow rate (Φ) is based on the dynamic viscosity (η) of water at 22 °C, η = 0.01 dyn∙s/cm^2^ and other parameters specific to the geometry of the system. Leukocyte-endothelial interactions were recorded for 5.5 min and firm leukocyte adhesion was quantified. Firm adhesion was defined by leukocytes that remained adhered on human brain endothelium in the field of view (FOV 640 × 480 μm) throughout the accumulation time, and, after increasing the flow to 1.5 dyn/cm^2^, cells were manually counted using Image J software in five or ten different FOVs randomly taked along the centreline of the channel. For quantification, all firmly adhered cells in the five or ten different FOVs of one indipendent experiment were counted, then the average was calculated. Data in the graphs are mean ± SEM of 3 to 5 independent experiments expressed as firmly adhered cells/FOV. Image acquisition was performed using an of an inverted fluorescence microscope (Olympus IX70, Tokyo, Japan) -X10 objective- controlled by the Image Pro Plus software (Media Cybernetics Inc. Bethesda, USA) using a Q-IMAGING *QICAM FAST* 1394 on a 12-bit camera (40 images/min).

### ELISA for membrane-associated adhesion molecules

Brain endothelial expression of VCAM1 (CD106), ICAM1 (CD54), ICAM2 (CD102) and E-selectin (CD62E) was measured by cell-surface ELISA performed as previously described^69^ using 2 μg/ml monoclonal anti-human mouse primary antibody against VCAM1 or ICAM1 or ICAM2 (R&D SYSTEMS, Abingdon, UK) or E-selectin (AbD SEROTEC Oxford, UK) and the corresponding secondary antibodies conjugated to horseradish peroxidase. The optical density (OD) was then measured using a FLUOstar Optima spectrometer (BMG LABTECH, Aylesbury, UK) at a wavelength of 450 nm. Absorbance from the wells with “blank” sample (without primary antibody) was subtracted from each of the corresponding treatment samples.

### Capture ELISA for secreted chemokines

Culture supernatants of confluent hCMEC/D3 cells were collected and frozen at −20 °C. For quantitative determination of chemokines, the human CCL7 and the human CCL2 Quantikine^®^ ELISA kits (R&D systems, Abingdon, UK) were used following the supplier’s protocols. The detection limits for CCL7 was 15.6 pg/ml and for CCL2 was 31.2 pg/ml. Signal saturation was observed at concentrations of >1000 or >2000 pg/ml for CCL7 or CCL2, respectively. Unstimulated hCMEC/D3 cell culture supernatants were first diluted in assay diluent at ratios of 1/3 (v/v) and 1/10 (v/v) for CCL7 and CCL2, respectively, while stimulated hCMEC/D3 cells culture supernatants were diluted in assay diluent at ratios of 1/3 (v/v) or 1/50 (v/v) for CCL7 and CCL2, respectively. The optical density (OD) was measured using a FLUOstar Optima spectrometer (BMG LABTECH) at a wavelength of 450 nm. Mean absorbance in the wells with “blank” sample (assay diluent only) was subtracted from the absorbance of each sample and the standards (known concentration of protein). The concentration of chemokines was determined by interpolation from the standard curve.

### Reverse transcription Real time-qPCR

For assessment of miR levels in cultured cells, total RNA was isolated from confluent cells using TRIzol^®^ Reagent (Invitrogen) following the manufacturer’s protocol. cDNA was generated from total RNA using a TaqMan High Capacity cDNA Reverse Transcription kit (Applied Biosystem, Life Technologies, Warrington, UK) with specific primers for miR-126, 126* and for small nuclear RNA U6, 002228-4427975, 000451-4427975 and 001973-4427975 respectively (Applied Biosystems Foster City, USA). RT^2^-qPCR was performed using the TaqMan MicroRNA assay (Applied Biosystem Life Technologies, Warrington, UK) with specific primers according to the manufacturer’s protocol.

Cellular miR levels were detected using the DNA Engine Opticon2 Real-Time System (MJ Research, St. Bruno, Canada) thermal cycler and Opticon Monitor software (MJ Research, St. Bruno, Canada) for data analysis. The relative amount of microRNA was calculated using the 2^−ΔΔCt^ (delta-delta Ct) method^70^ and normalized with the commercially available internal control, the small nuclear RNA U6. The results of microRNA relative levels in treated and/or transfected hCMEC/D3 cells were expressed as fold increase over microRNA levels in unstimulated and control transfected hCMEC/D3 cells.

### Immunocytochemistry

hCMEC/D3 cell monolayes in IbidÍ chambers were fixed for 10 min with 4% *p*-formaldehyde and then incubated with blocking solution, 5% goat serum. VCAM1 expression was detected using monoclonal anti-human mouse VCAM1 primary antibody (R&D SYSTEMS, Abingdon, UK) followed by the secondary antibodies Alexa Fluor^®^ 488 Goat Anti-Mouse IgG. Cells were mounted using mounting media with DAPI. Pictures were acquired with a Zeiss microscope using axiophot prism filter set (λ_ex_ − λ_em_: blue 450–490 nm, green 395–440 nm) with X40 objective. The signal was quantified using the software, Image J (Java-based image processing program developed at the National Institutes of Health).

### Bioinformatic analysis

Predicted mRNA targets for miR-126 and -126* were identified using eight well known microRNA target prediction programs/databases: Targetscan v5.0 (http://www.targetscan.org/), Miranda (http://www.microrna.org/microrna/home.do), Pictar (http://pictar.mdc-berlin.de/), Microcosm (http://www.ebi.ac.uk/enright-srv/microcosm/cgibin/targets/v5/search.pl), Tarbase (http://diana.cslab.ece.ntua.gr/tarbase/), DianaLab Microt (http://diana.cslab.ece.ntua.gr/microT/), Diana Lab (http://diana.cslab.ece.ntua.gr/mirgen/), Target Miner (http://www.isical.ac.in/~bioinfo_miu/), MirDB (http://mirdb.org/miRDB/).

To generate the heatmap for miR-126 and miR-126*, raw data from public database Gene Expression Omnibus (GEO)-Geo accession GSE21350 were normalized and transformed into z-score data. TM4: MeV (Multiple Experiment Viewer), a Java tool for genomic data analysis was used to generate the heatmap with the z-score data. For each treatment, three replicates were analized.

### Statistical analysis

All data are presented as mean ± SEM (standard error of the mean) from a number of independent experiments (n) with replicates specified in each legend. *P* values were calculated using unpaired Student’s *t* tests. Statistically significant differences are presented as probability levels of *P *<* *0.05 (*), *P* < 0.01 (**), *P* < 0.001 (***). Calculations and figures were performed using the statistical software GraphPad Prism 5 (GraphPad Software).

## Additional Information

**How to cite this article**: Cerutti, C. *et al*. MiR-126 and miR-126* regulate shear-resistant firm leukocyte adhesion to human brain endothelium. *Sci. Rep.* **7**, 45284; doi: 10.1038/srep45284 (2017).

**Publisher's note:** Springer Nature remains neutral with regard to jurisdictional claims in published maps and institutional affiliations.

## Supplementary Material

Supplementary Information

Supplementary Video S1

Supplementary Video S2

## References

[b1] Engelhardt B. & Ransohoff R. M. Capture, crawl, cross: the T cell code to breach the blood-brain barriers. Trends Immunol 33, 579–589, doi: 10.1016/j.it.2012.07.004 (2012). 22926201

[b2] Ousman S. S. & Kubes P. Immune surveillance in the central nervous system. Nat Neurosci 15, 1096–1101, doi: 10.1038/nn.3161 (2012). 22837040 PMC7097282

[b3] McFarland H. F. & Martin R. Multiple sclerosis: a complicated picture of autoimmunity. Nat Immunol 8, 913–919, doi: 10.1038/ni1507 (2007). 17712344

[b4] Owens T., Bechmann I. & Engelhardt B. Perivascular spaces and the two steps to neuroinflammation. J Neuropathol Exp Neurol 67, 1113–1121, doi: 10.1097/NEN.0b013e31818f9ca8 (2008). 19018243

[b5] Hughes C. C., Male D. K. & Lantos P. L. Adhesion of lymphocytes to cerebral microvascular cells: effects of interferon-gamma, tumour necrosis factor and interleukin-1. Immunology 64, 677–681 (1988). 3139550 PMC1384990

[b6] Sharief M. K. & Hentges R. Association between tumor necrosis factor-alpha and disease progression in patients with multiple sclerosis. N Engl J Med 325, 467–472, doi: 10.1056/nejm199108153250704 (1991). 1852181

[b7] Sospedra M. & Martin R. Immunology of multiple sclerosis. Annual review of immunology 23, 683–747, doi: 10.1146/annurev.immunol.23.021704.115707 (2005). 15771584

[b8] Engelhardt B. Regulation of immune cell entry into the central nervous system. Results Probl Cell Differ 43, 259–280 (2006). 17068976 10.1007/400_020

[b9] Lucchinetti C. F., Parisi J. & Bruck W. The pathology of multiple sclerosis. Neurologic clinics 23, 77–105, vi, doi: 10.1016/j.ncl.2004.09.002 (2005). 15661089

[b10] McManus C. . MCP-1, MCP-2 and MCP-3 expression in multiple sclerosis lesions: an immunohistochemical and *in situ* hybridization study. J Neuroimmunol 86, 20–29, doi: 10.1016/S0165-5728(98)00002-2 (1998). 9655469

[b11] Olson T. S. & Ley K. Chemokines and chemokine receptors in leukocyte trafficking. Am J Physiol Regul Integr Comp Physiol 283, R7–28, doi: 10.1152/ajpregu.00738.2001 (2002). 12069927

[b12] Cerutti C. . MicroRNA-155 contributes to shear-resistant leukocyte adhesion to human brain endothelium *in vitro*. Fluids Barriers CNS 13, 8, doi: 10.1186/s12987-016-0032-3 (2016). 27246706 PMC4888311

[b13] Bartel D. P. MicroRNAs: target recognition and regulatory functions. Cell 136, 215–233, doi: 10.1016/j.cell.2009.01.002 (2009). 19167326 PMC3794896

[b14] Thamilarasan M., Koczan D., Hecker M., Paap B. & Zettl U. K. MicroRNAs in multiple sclerosis and experimental autoimmune encephalomyelitis. Autoimmun Rev 11, 174–179, doi: 10.1016/j.autrev.2011.05.009 (2012). 21621006

[b15] Rom S., Dykstra H., Zuluaga-Ramirez V., Reichenbach N. L. & Persidsky Y. miR-98 and let-7g* protect the blood-brain barrier under neuroinflammatory conditions. J Cereb Blood Flow Metab 35, 1957–1965, doi: 10.1038/jcbfm.2015.154 (2015). 26126865 PMC4671116

[b16] Wu D. . Brain endothelial miR-146a negatively modulates T-cell adhesion through repressing multiple targets to inhibit NF-kappaB activation. J Cereb Blood Flow Metab 35, 412–423, doi: 10.1038/jcbfm.2014.207 (2015). 25515214 PMC4348377

[b17] Fish J. E. . miR-126 regulates angiogenic signaling and vascular integrity. Dev Cell 15, 272–284, doi: 10.1016/j.devcel.2008.07.008 (2008). 18694566 PMC2604134

[b18] Harris T. A., Yamakuchi M., Ferlito M., Mendell J. T. & Lowenstein C. J. MicroRNA-126 regulates endothelial expression of vascular cell adhesion molecule 1. Proc Natl Acad Sci USA 105, 1516–1521, doi: 10.1073/pnas.0707493105 (2008). 18227515 PMC2234176

[b19] McCall M. N. . MicroRNA profiling of diverse endothelial cell types. BMC Med Genomics 4, 78, doi: 10.1186/1755-8794-4-78 (2011). 22047531 PMC3223144

[b20] Wang S. . The endothelial-specific microRNA miR-126 governs vascular integrity and angiogenesis. Dev Cell 15, 261–271, doi: 10.1016/j.devcel.2008.07.002 (2008). 18694565 PMC2685763

[b21] Taverna S. . Exosomal shuttling of miR-126 in endothelial cells modulates adhesive and migratory abilities of chronic myelogenous leukemia cells. Mol Cancer 13, 169, doi: 10.1186/1476-4598-13-169 (2014). 25015105 PMC4105877

[b22] Yang J. S. . Widespread regulatory activity of vertebrate microRNA* species. Rna 17, 312–326, doi: 10.1261/rna.2537911 (2011). 21177881 PMC3022280

[b23] Huang X. . Regulated expression of microRNAs-126/126* inhibits erythropoiesis from human embryonic stem cells. Blood 117, 2157–2165, doi: 10.1182/blood-302711 (2010) (2011). 21163928 PMC3062325

[b24] Schober A. . MicroRNA-126-5p promotes endothelial proliferation and limits atherosclerosis by suppressing Dlk1. Nat Med 20, 368–376, doi: 10.1038/nm.3487 (2014). 24584117 PMC4398028

[b25] Meister J. & Schmidt M. H. miR-126 and miR-126*: new players in cancer. ScientificWorldJournal 10, 2090–2100, doi: 10.1100/tsw.2010.198 (2010). 20953557 PMC5763667

[b26] Musiyenko A., Bitko V. & Barik S. Ectopic expression of miR-126*, an intronic product of the vascular endothelial EGF-like 7 gene, regulates prostein translation and invasiveness of prostate cancer LNCaP cells. J Mol Med (Berl) 86, 313–322, doi: 10.1007/s00109-007-0296-9 (2008). 18193184 PMC3263384

[b27] Felli N. . miR-126&126* Restored Expressions Play a Tumor Suppressor Role by Directly Regulating ADAM9 and MMP7 in Melanoma. PLoS One 8, e56824; doi: 10.1371/pone.0056824 (2013). 23437250 PMC3578857

[b28] Zhang Y. . miR-126 and miR-126(*) repress recruitment of mesenchymal stem cells and inflammatory monocytes to inhibit breast cancer metastasis. Nat Cell Biol 15, 284–294, doi: 10.1038/ncb2690 (2013). 23396050 PMC3672398

[b29] Poissonnier L., Villain G., Soncin F. & Mattot V. miR126-5p repression of ALCAM and SetD5 in endothelial cells regulates leucocyte adhesion and transmigration. Cardiovasc Res 102, 436–447, doi: 10.1093/cvr/cvu040 (2014). 24562769

[b30] Weksler B. B. . Blood-brain barrier-specific properties of a human adult brain endothelial cell line. FASEB J. 04-3458fje, doi: 10.1096/fj.04-3458fje (2005). 16141364

[b31] Wong D. & Dorovini-Zis K. Regualtion by cytokines and lipopolysaccharide of E-selectin expression by human brain microvessel endothelial cells in primary culture. J Neuropathol Exp Neurol 55, 225–235 (1996). 8786381 10.1097/00005072-199602000-00011

[b32] de Fougerolles A. R., Stacker S. A., Schwarting R. & Springer T. A. Characterization of ICAM-2 and evidence for a third counter-receptor for LFA-1. J Exp Med 174, 253–267 (1991). 1676048 10.1084/jem.174.1.253 PMC2118873

[b33] Nortamo P. . The expression of human intercellular adhesion molecule-2 is refractory to inflammatory cytokines. Eur J Immunol 21, 2629–2632, doi: 10.1002/eji.1830211049 (1991). 1680706

[b34] McLaughlin F. . Tumor necrosis factor (TNF)-alpha and interleukin (IL)-1beta down-regulate intercellular adhesion molecule (ICAM)-2 expression on the endothelium. Cell Adhes Commun 6, 381–400 (1998). 10223354 10.3109/15419069809109147

[b35] Lopez-Ramirez M. A. . MicroRNA-155 negatively affects blood-brain barrier function during neuroinflammation. Faseb j 28, 2551–2565, doi: 10.1096/fj.13-248880 (2014). 24604078

[b36] Battistini L. . CD8+ T cells from patients with acute multiple sclerosis display selective increase of adhesiveness in brain venules: a critical role for P-selectin glycoprotein ligand-1. Blood 101, 4775–4782, doi: 10.1182/blood-2002-10-3309 (2003). 12595306

[b37] Rudolph H. . Postarrest stalling rather than crawling favors CD8+ over CD4+ T-cell migration across the blood-brain barrier under flow *in vitro*. European journal of immunology, doi: 10.1002/eji.201546251 (2016). PMC5113696 27338806

[b38] Sun C. . IRF-1 and miRNA126 modulate VCAM-1 expression in response to a high-fat meal. Circ Res 111, 1054–1064, doi: 10.1161/circresaha.112.270314 (2012). 22874466 PMC3810165

[b39] Asgeirsdottir S. A. . MicroRNA-126 contributes to renal microvascular heterogeneity of VCAM-1 protein expression in acute inflammation. Am J Physiol Renal Physiol 302, F1630–1639, doi: 10.1152/ajprenal.00400.2011 (2012). 22419694

[b40] Salvucci O. . MicroRNA126 contributes to granulocyte colony-stimulating factor-induced hematopoietic progenitor cell mobilization by reducing the expression of vascular cell adhesion molecule 1. Haematologica 97, 818–826, doi: 10.3324/haematol.2011.056945 (2012). 22271895 PMC3366645

[b41] Arner E. . Adipose tissue microRNAs as regulators of CCL2 production in human obesity. Diabetes 61, 1986–1993, doi: 10.2337/db11-1508 (2012). 22688341 PMC3402332

[b42] Zhang Y. . miR-126 and miR-126* repress recruitment of mesenchymal stem cells and inflammatory monocytes to inhibit breast cancer metastasis. Nat Cell Biol 15, 284–294, doi: 10.1038/ncb2690 (2013). 23396050 PMC3672398

[b43] Chen J. . MiR-126 Contributes to Human Umbilical Cord Blood Cell-Induced Neurorestorative Effects After Stroke in Type-2 Diabetic Mice. Stem Cells 34, 102–113, doi: 10.1002/stem.2193 (2016). 26299579 PMC4713352

[b44] Zernecke A. . Delivery of microRNA-126 by apoptotic bodies induces CXCL12-dependent vascular protection. Sci Signal 2, ra81, doi: 10.1126/scisignal.2000610 (2009). 19996457

[b45] Minagar A. & Alexander J. S. Blood-brain barrier disruption in multiple sclerosis. Mult Scler 9, 540–549 (2003). 14664465 10.1191/1352458503ms965oa

[b46] Takeshita Y. & Ransohoff R. M. Inflammatory cell trafficking across the blood-brain barrier: chemokine regulation and *in vitro* models. Immunol Rev 248, 228–239, doi: 10.1111/j.1600-065X.2012.01127.x (2012). 22725965 PMC3383666

[b47] Lopez-Ramirez M. A. . Role of caspases in cytokine-induced barrier breakdown in human brain endothelial cells. J Immunol 189, 3130–3139, doi: 10.4049/jimmunol.1103460 (2012). 22896632

[b48] Soilu-Hanninen M. . Progressive multifocal leukoencephalopathy as a complication of natalizumab therapy. Duodecim 129, 765–770 (2013). 23720945

[b49] Lim L. P. . Microarray analysis shows that some microRNAs downregulate large numbers of target mRNAs. Nature 433, 769–773, doi: 10.1038/nature03315 (2005). 15685193

[b50] Guo H., Ingolia N. T., Weissman J. S. & Bartel D. P. Mammalian microRNAs predominantly act to decrease target mRNA levels. Nature 466, 835–840, doi: 10.1038/nature09267 (2010). 20703300 PMC2990499

[b51] Baek D. . The impact of microRNAs on protein output. Nature 455, 64–71, doi: 10.1038/nature07242 (2008). 18668037 PMC2745094

[b52] Inui M., Martello G. & Piccolo S. MicroRNA control of signal transduction. Nat Rev Mol Cell Biol 11, 252–263, doi: 10.1038/nrm2868 (2010). 20216554

[b53] Oglesby I. K. . miR-126 is downregulated in cystic fibrosis airway epithelial cells and regulates TOM1 expression. J Immunol 184, 1702–1709, doi: 10.4049/jimmunol.0902669 (2010). 20083669

[b54] Bai Y. . MicroRNA-126 inhibits ischemia-induced retinal neovascularization via regulating angiogenic growth factors. Exp Mol Pathol 91, 471–477, doi: 10.1016/j.yexmp.2011.04.016 (2011). 21586283

[b55] Yanaihara N. . Unique microRNA molecular profiles in lung cancer diagnosis and prognosis. Cancer Cell 9, 189–198, doi: 10.1016/j.ccr.2006.01.025 (2006). 16530703

[b56] Harris T. A., Yamakuchi M., Kondo M., Oettgen P. & Lowenstein C. J. Ets-1 and Ets-2 regulate the expression of microRNA-126 in endothelial cells. Arterioscler Thromb Vasc Biol 30, 1990–1997, doi: 10.1161/atvbaha.110.211706 (2010). 20671229 PMC3121560

[b57] Goetze S. . TNFalpha induces expression of transcription factors c-fos, Egr-1, and Ets-1 in vascular lesions through extracellular signal-regulated kinases 1/2. Atherosclerosis 159, 93–101 (2001). 11689211 10.1016/s0021-9150(01)00497-x

[b58] Russell L. & Garrett-Sinha L. A. Transcription factor Ets-1 in cytokine and chemokine gene regulation. Cytokine 51, 217–226, doi: 10.1016/j.cyto.2010.03.006 (2010). 20378371

[b59] Hu S., Zhu W., Zhang L. F., Pei M. & Liu M. F. MicroRNA-155 broadly orchestrates inflammation-induced changes of microRNA expression in breast cancer. Cell research 24, 254–257, doi: 10.1038/cr.2013.137 (2014). 24080728 PMC3915901

[b60] Westmuckett A. D., Thacker K. M. & Moore K. L. Tyrosine sulfation of native mouse Psgl-1 is required for optimal leukocyte rolling on P-selectin *in vivo*. PLoS One 6, e20406, doi: 10.1371/journal.pone.0020406 (2011). 21633705 PMC3102115

[b61] Nishimura Y. . Human P-selectin glycoprotein ligand-1 is a functional receptor for enterovirus 71. Nat Med 15, 794–797, doi: 10.1038/nm.1961 (2009). 19543284

[b62] Ko Y. C. . Endothelial CD200 is heterogeneously distributed, regulated and involved in immune cell-endothelium interactions. J Anat 214, 183–195, doi: 10.1111/j.1469-7580.2008.00986.x (2009). 19166481 PMC2667927

[b63] Cayrol R. . Activated leukocyte cell adhesion molecule promotes leukocyte trafficking into the central nervous system. Nat Immunol 9, 137–145, doi: 10.1038/ni1551 (2008). 18157132

[b64] Broderick J. A. & Zamore P. D. MicroRNA therapeutics. Gene Ther 18, 1104–1110; doi: 10.1038/gt.2011.50 (2011). 21525952 PMC3237828

[b65] Collison A. . Altered expression of microRNA in the airway wall in chronic asthma: miR-126 as a potential therapeutic target. BMC Pulm Med 11, doi: 10.1186/1471-2466-11-29 (2011). PMC3116478 21605405

[b66] Kota J. . Therapeutic microRNA delivery suppresses tumorigenesis in a murine liver cancer model. Cell 137, 1005–1017; doi: 10.1016/j.cell.2009.04.021 (2009). 19524505 PMC2722880

[b67] Endo-Takahashi Y. . Systemic delivery of miR-126 by miRNA-loaded Bubble liposomes for the treatment of hindlimb ischemia. Sci Rep 4, 3883, doi: 10.1038/srep03883 (2014). 24457599 PMC3900923

[b68] Ma S. . E-selectin-targeting delivery of microRNAs by microparticles ameliorates endothelial inflammation and atherosclerosis. Sci Rep 6, 22910, doi: 10.1038/srep22910 (2016). 26956647 PMC4783714

[b69] Male D. K., Pryce G. & Hughes C. C. Antigen presentation in brain: MHC induction on brain endothelium and astrocytes compared. Immunology 60, 453–459 (1987). 3106198 PMC1453249

[b70] Livak K. J. & Schmittgen T. D. Analysis of relative gene expression data using real-time quantitative PCR and the 2(-Delta Delta C(T)) Method. Methods 25, 402–408, doi: 10.1006/meth.2001.1262 (2001). 11846609

