# Supplementary material for: MiR-126 and miR-126* regulate shear-resistant firm leukocyte adhesion to human brain endothelium
Source: Sci Rep. 2017 Mar 30;7:45284. doi: 10.1038/srep45284 (PMC5372244; doi:10.1038/srep45284)
Supplement: Supplementary Information [file srep45284-s1.pdf]

MiR-126 and miR-126* regulate shear-resistant firm leukocyte adhesion to human brain endothelium

Camilla Cerutti[1,5], Laura J Edwards[2,6], Helga E de Vries[3], Basil Sharrack[4], David K. Male[1] and Ignacio A Romero[1]

SUPPLEMENTARY DATA

Figures S1, S2, S3, S4

Video S1, S2

Supplementary Material and Methods

Figure S1

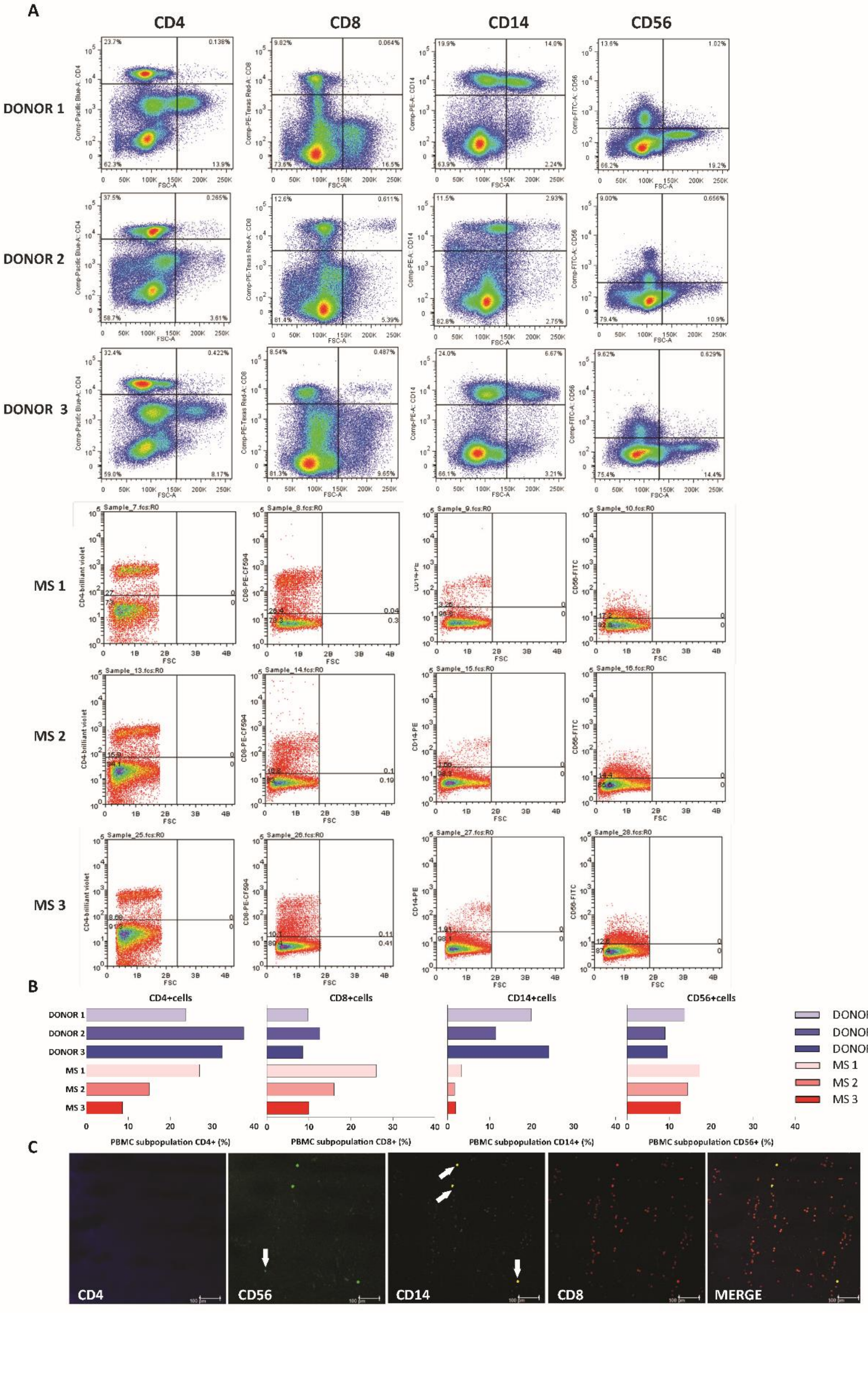
A
CD4
CD8
CD14
CD56
DONOR 1
DONOR 2
DONOR 3
MS 1
MS 2
MS 3
B
CD4+cells
CD8+cells
CD14+cells
CD56+cells
PBMC subpopulation CD4+ (%)
PBMC subpopulation CD8+ (%)
PBMC subpopulation CD14+ (%)
PBMC subpopulation CD56+ (%)
DONOR 1
DONOR 2
DONOR 3
MS 1
MS 2
MS 3
C
CD4
CD56
CD14
CD8
MERGE

Supplementary Fig. 1: PBMC characterisation (**A**) Quantification of monocyte, T cell and NK cell subpopulations by FACS in healthy donors and MS patient-derived PBMC by conjugated mouse anti-human CD4-brilliant violet, CD8-PE-CF594, CD14-PE and CD56-FITC primary antibodies with relative (**B**) percentages of CD4, CD8, CD14 and CD56 positive cells in healthy donor and MS patient-derived PBMC samples. (**C**) Identification of monocyte, T cell and NK cell subpopulations in firmly adhered MS patients derived PBMC to hCMEC/D3 cells transfected with SCRAMBLED Pre-miR and stimulated with a combination of cytokines (TNFα + IFNγ) at 1 ng/ml, by conjugated mouse anti-human CD4-brilliant violet, CD8-PE-CF594, CD14-PE and CD56-FITC primary antibodies. Confocal images, Bar -100μm.

Figure S2

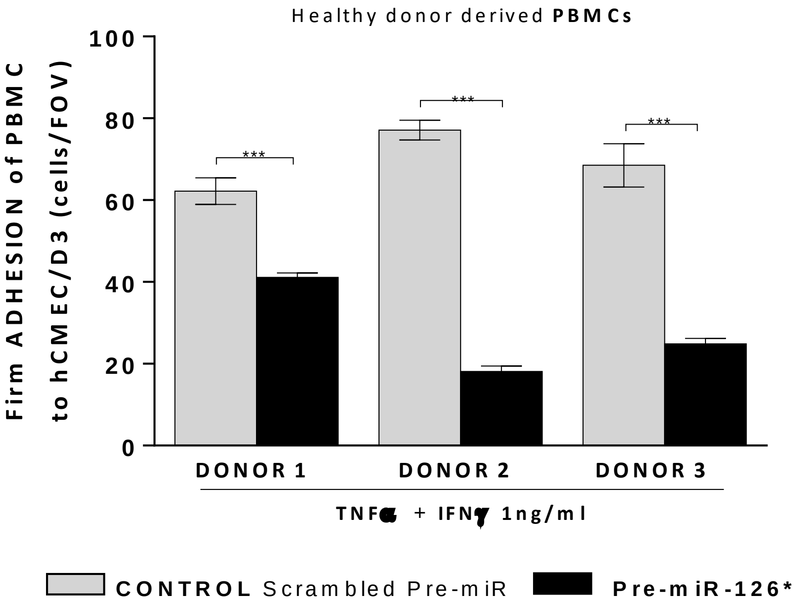

Supplementary Figure 2: miR-126* upregulation decreases healthy donor PBMC firm adhesion to hCMEC/D3 cells. hCMEC/D3 cells were transfected with control Scrambled Pre-miR (grey) or with Pre-miR-126* (black) followed by treatment with cytokines (TNFα + IFNγ, 1 ng/ml) for 24 h. Numbers of shear-resistant firmly adhered healthy donor PBMC to cytokine-stimulated hCMEC/D3 monolayers per field of view (FOV) (640 x 480 μm). Data are mean ±SEM. ****P*<0.001.

Figure S3

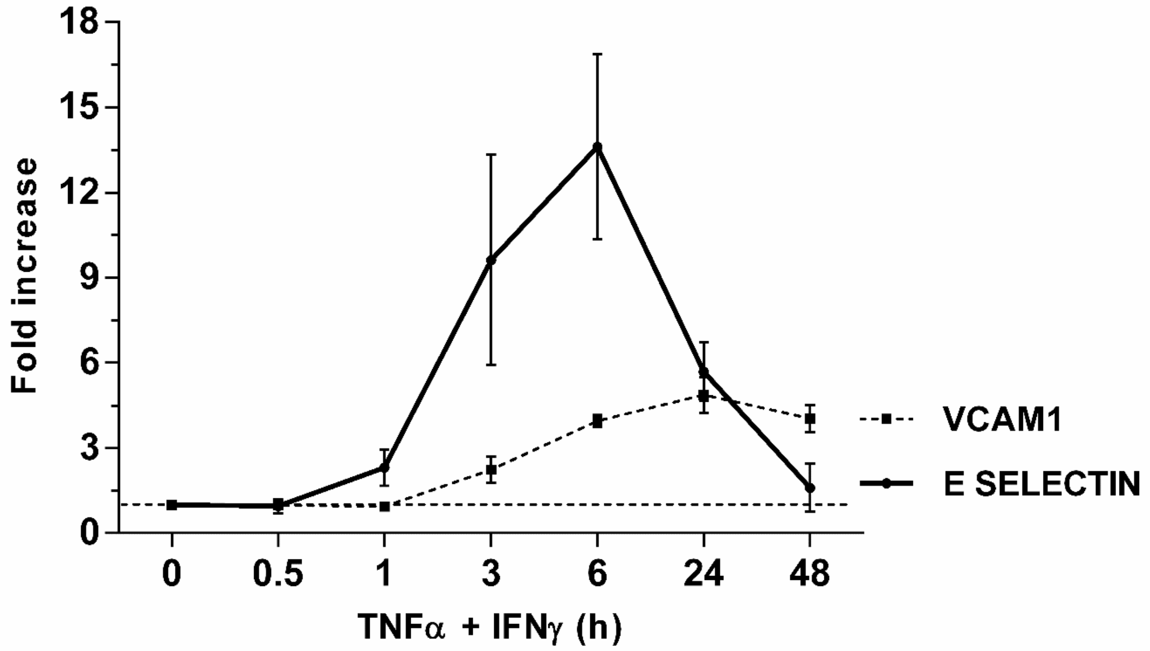

Supplementary Fig. 3: **Combination of cytokines (TNFα and IFNγ) increases VCAM1 and E-selectin expression on hCMEC/D3 cells.** Confluent hCMEC/D3 cell monolayers were treated with TNFα and IFNγ in combination for different times (0, 0.5, 1, 3, 6, 24 and 48 h). Anti-human-E-selectin and -VCAM1 monoclonal antibodies were used to detect E-selectin and VCAM1 expression levels by ELISA. Experiments were carried out three times with three replicates. Data are mean ±SEM.

Figure S4

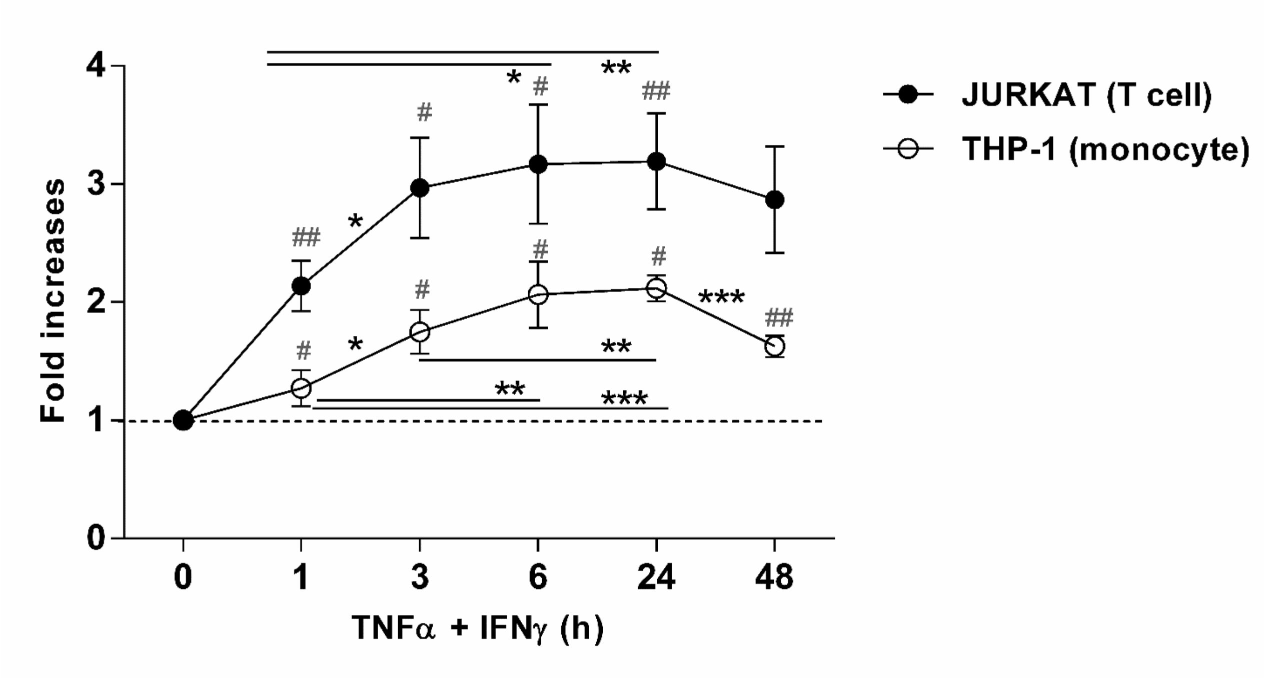

Supplementary Fig. 4: Combination of cytokines (TNFα and IFNγ) increases adhesion of leukocytes to hCMEC/D3 cells in a time-dependent manner. Confluent hCMEC/D3 cell monolayers were treated with TNFα and IFNγ in combination at different times (0, 1, 3, 6, 24 and 48 h). Fluorescence of adhered THP-1 or Jurkat cells to hCMEC/D3 cell monolayers was quantified at λ_{ex}= 485nm and λ_{em}= 525nm. Data are normalized to leukocyte adhesion levels on unstimulated hCMEC/D3 cell monolayers. Experiments were carried out three times with six replicates each. Data are mean ±SEM (*,$^{\#}p$<0.05**,$^{\#\#}p$<0.01, ***,$^{\#\#\#}p$<0.001, $^{\#}$ significantly different vs. unstimulated cells, * significantly different between cytokine doses or time points).

Supplemental Video 1: Shear-resistant firm adhesion of multiple sclerosis-derived PBMCs to CONTROL Scrambled-Pre-miR transfected- and stimulated-hCMEC/D3 cells. Live cell imaging was used to record both the endothelial monolayer and multiple sclerosis-derived PBMCs by light microscopy. Multiple sclerosis-derived PBMCs were pulled through the channel over hCMEC/D3 monolayers under low shear (0.5 dyn/cm^2). After 5 min, flow shear stress was increased (1.5 dyn/cm^2) to challenge non-firmly adherent PBMCs. The number of arrested PBMCs constantly increased during the accumulation phase (small rounded white cells) and only the one that remained stationary on the endothelial monolayer were manually counted in ten different FOVs along the channel of Ibidi μ-Slide VI. Example of a FOV picture with shear-resistant firmly adhered multiple sclerosis-derived PBMC to hCMEC/D3 cells to count is depicted in Fig. 3. Objective 10x, at 43 images per min, field of view 640 µm × 480 µm, recording time 5.5 min.

Supplemental Video 2: Shear-resistant firm adhesion of multiple sclerosis-derived PBMCs to Pre-miR-126 transfected- and stimulated-hCMEC/D3 cells. Live cell imaging was used to record both the endothelial monolayer and multiple sclerosis-derived PBMCs by light microscopy. Multiple sclerosis-derived PBMCs were pulled through the channel over hCMEC/D3 monolayers under low shear (0.5 dyn/cm^2). After 5 min, flow shear stress was increased (1.5 dyn/cm^2) to challenge non-firmly adherent PBMCs. The number of arrested PBMCs constantly increased during the accumulation phase (small rounded white cells) and only the one that remained stationary on the endothelial monolayer were manually counted in ten different FOVs along the channel of Ibidi μ-Slide VI. Example of a FOV picture with shear-resistant firmly adhered multiple sclerosis-derived PBMC to hCMEC/D3 cells to count is depicted in Fig. 3. Objective 10x, at 43 images per min, field of view 640 μm × 480 μm, recording time 5.5 min.

Supplementary Material and Methods

Flow cytometry analysis

For characterization of subpopulations in isolated PBMC, cells were thawed in warm RPMI and counted. PBMC were resuspended at 0.5×10^6 cells/ml in phosphate buffered saline (PBS) and placed in FACS tubes (1×10^6 cells/2ml/tube), then spun down for 10 min at 254 x *g*. The cell pellet was resuspended in 50 μl of PBS (1×10^6/100μl) and incubated with fluorescently labelled primary antibody for 30 min at 4 °C at the concentrations indicated in Table 2.4. Followed by two washes in PBA (1/200 (v/v) 20% sodium azide + 1/60 (v/v) 30% bovine serum albumin (BSA) in PBS), PBMC were resuspended in 400 μl in a solution of 0.5% methanol in PBS and stored at 4 °C until analysis using flow cytometry with Becton Dickinson FacsCanto II (BD, Oxford, UK). Data were analyzed using FACSDiva software (BD, Oxford, UK). Results are expressed as percentage of positive cells.

Static leukocyte adhesion assay

Cell loading with cell tracker™ green CMFDA (5-chloromethylfluorescein diacetate) was carried out as indicated by the manufacturer (Invitrogen, Paisley, UK). Jurkat or THP-1cells were labelled 5 mM CMFDA or left unlabelled in RPMI media without serum and antibiotics for 30 min at 37 °C. Leukocytes were then centrifuged at (190 x *g*) and re-suspended in RPMI media without serum or antibiotics for 30 min at 37° C. Fluorescently labelled leukocytes in EBM-2 complete media ($2x10^5$ leukocytes cells/well) were added onto cytokine-treated hCMEC/D3 cells for 1 h at 37 °C. After washing three times with 200 μl HBSS, the fluorescence of leukocytes remaining adherent to the hCMEC/D3 monolayer was measured using a FLUOstar Optima fluorescence plate reader (BMG LABTECH) as above. This assay was adapted from static assays used previously (Solito, Romero et al. 2000; Hisano, Namba et al. 2005). The software Optima version 2.00R3 (BMG LABTECH, Tampa, USA) was used to acquire and analyse the data. The percentage of adherent leukocytes was calculated using the following formula:

$$\frac{\textbf{Fluorescence signal in experimental well - fluorescence signal blank well}}{\textbf{Fluorescence signal in input well - fluorescence signal input blank well}} \quad \textbf{X 100}$$

Where the experimental wells were hCMEC/D3 cells plus adhered leukocytes in EBM-2 media and the blank wells were hCMEC/D3 cells in EBM-2 media only. The input wells were $2x10^5$ leukocytes and the input blank wells were HBSS. A standard curve using the fluorescence intensities of labelled leukocyte suspensions with $2x10^3$, $2x10^4$ and $2x10^5$ cells in HBSS corresponding to 1, 10 and 100% input, respectively, was then plotted to determine the % of adherent leukocytes.
